# Comment on “Puerarin Improves Diabetic Aorta Injury by Inhibiting NADPH Oxidase-Derived Oxidative Stress in STZ-Induced Diabetic Rats”

**DOI:** 10.1155/2016/7302620

**Published:** 2016-08-23

**Authors:** Qiang Xie, Jian Zhong, Jun Li

**Affiliations:** ^1^PET/CT Center, Anhui Provincial Hospital, Hefei 230001, China; ^2^Anhui Joyfar Pharmaceutical Co., Ltd., Kexuedadao Road, Hefei 230000, China; ^3^School of Pharmacy, Anhui Medical University, Meishan Road, Hefei 230032, China; ^4^Institute for Liver Diseases, Anhui Medical University, Hefei 230032, China; ^5^Anhui Key Laboratory of Bioactivity of Natural Products, Anhui Medical University, Hefei 230032, China

We read with great interest the article showing that streptozotocin- (STZ-) induced diabetic rats given puerarin can significantly reduce the serum levels of insulin, glycated hemoglobin, PGE2, endothelin, H_2_O_2_, and NO, improve the pathological alterations, and inhibit the expression of ICAM-1, LOX-1, and NOX2 [1]. In addition, puerarin strongly reduced the number of cells showing positive staining for ICAM-1, NOX2, NOX4, and NF-*κ*B p65. Interestingly, Li et al. also showed that STZ-induced diabetes mellitus and aorta lesion can be improved by treatment with puerarin, where puerarin is able to downregulate the levels of cholesterol, low density lipoprotein, P-selectin, oxidative low density lipoprotein, and the expression of VCAM in the aorta which were significantly inhibited [2]. These data indicated that puerarin has played a certain role in preventing aorta or vessels by inhibiting expression of adhesion molecules.

Diabetes is a chronic disease characterized by hyperglycemia. It results in numerous chronic microvascular and macrovascular complications, such as nephropathy and atherosclerosis. These chronic complications are the major causes of the downregulated quality of life among diabetics, elevated burden to the health care system. Thus, inhibiting and alleviating these complications is helpful in diabetes treatment. Puerarin is the main bioactive component of* getongtongluo* capsule, extracted from the Chinese herb lobed kudzuvine root [3]. Puerarin has been recognized to inhibit high glucose-induced upregulation of H3K4 di- and trimethylation (H3K4me2/3) on the MCP-1 gene promoter, possessing a therapeutic potential in diabetes-induced vascular injuries [4]. It also significantly inhibited rat vascular smooth muscle cells proliferation, ROS generation, and NADPH oxidase activity induced by high glucose treatment [5]. Interestingly, neointimal formation of obese rats evoked by balloon injury was attenuated by the administration of puerarin. Moreover, insulin resistance was established by palmitate stimulation in the endothelium, and Huang et al. [6] showed that palmitate stimulation evoked inflammatory response in endothelial cells but puerarin inhibited IKK*β*/NF-*κ*B activation and decreased TNF-*α*, IL-6 production, and insulin-mediated NO generation. In addition, rats incubated with high glucose decreased the vascular contraction responses to phenylephrine and relaxation response to acetylcholine, but treatment with puerarin inhibited the high glucose-induced vasoconstriction and vasodilation dysfunction and increased the HO-1 protein expression and HO activity of thoracic aorta [7].

Collectively, the available evidence suggests a potential role that puerarin plays in aorta or vessels improvement, especially in diabetes. However, further studies are needed to comprehensively explore the role of puerarin in diabetes and its complications, and the application of these therapeutic agents should be discussed in humans though available evidence is mainly determined in animals.
